# Safety, Quality, and Processing of Fruits and Vegetables

**DOI:** 10.3390/foods8110569

**Published:** 2019-11-13

**Authors:** Urszula Tylewicz, Silvia Tappi, Malgorzata Nowacka, Artur Wiktor

**Affiliations:** 1 Department of Agricultural and Food Sciences, University of Bologna, P.zza Goidanich 60, 47521 Cesena, Italy; 2 Interdepartmental Centre for Agri-Food Industrial Research, University of Bologna, Via Quinto Bucci 336, 47521 Cesena, Italy; silvia.tappi2@unibo.it; 3 Department of Food Engineering and Process Management, Faculty of Food Sciences, Warsaw University of Life Sciences, Nowoursynowska 159c, 02-776 Warsaw, Poland; malgorzata_nowacka@sggw.pl (M.N.); artur_wiktor@sggw.pl (A.W.)

**Keywords:** fruit, vegetable, safety, quality, emerging technologies, unconventional processing

## Abstract

Nowadays, one of the main objectives of the fruit and vegetable industry is to develop innovative novel products with high quality, safety, and optimal nutritional characteristics in order to respond with efficiency to the increasing consumer expectations. Various emerging, unconventional technologies (e.g., pulsed electric field, pulsed light, ultrasound, high pressure, and microwave drying) enable the processing of fruits and vegetables, increasing their stability while preserving their thermolabile nutrients, flavour, texture, and overall quality. Some of these technologies can also be used for waste and by-product valorisation. The application of fast noninvasive methods for process control is of great importance for the fruit and vegetable industry. The following Special Issue “Safety, Quality, and Processing of Fruits and Vegetables” consists of 11 papers, which provide a high-value contribution to the existing knowledge on safety aspects, quality evaluation, and emerging processing technologies for fruits and vegetables.

In the last few years, consumers have become more exigent and demand high-quality and convenient food products with natural flavours and taste, free from additives and preservatives [1]. Therefore, the challenge for the fruit and vegetable industry is to develop such products, taking into account quality and safety aspects along with consumer acceptance. Emerging, unconventional processing of fruit and vegetables is more and more studied in order to develop products rich in bioactive compounds, paying attention at the same time to waste and by-product valorisation [2,3,4]. This Special Issue “Safety, Quality, and Processing of Fruits and Vegetables” gives an overview of the application of emerging, unconventional technologies to obtain high-quality fruit juice, semi-dried and dried products, waste valorisation, and process control. It also provides some insights into principles and fundamentals of nonthermal technologies.

The importance of the quality standards for potatoes intended for the processing industry is explained by Wayumba et al. [5]. This study was designed with the purpose of identifying specialized potato clones with acceptable qualities for processing chips, as compared to two selected control varieties, Dubaek and Superior. From this study, the authors concluded that for quality processing of potato chips, clones with combined traits of high dry matter, low levels of glycoalkaloids and reducing sugars, should be used as raw materials along with the acceptable chip colour [5]. Starch is the major component in potato, that contributes to its nutritional and technological quality. Different food processing techniques including boiling, cooling, reheating, conventional frying, and air frying have been shown to change the digestibility of starch. In the paper by Abduh et al. [6], the effect of emerging processing using pulsed electric field (PEF)—usually used for structure modification in fruit and vegetables—on the properties of starch in potatoes was investigated, showing that PEF did not change the properties of starch within the potatoes, but it narrowed the temperature range of gelatinisation and reduced the digestibility of starch collected from the processing medium. Therefore, this study confirms that, when used as a processing aid for potato, PEF does not result in detrimental effects on the properties of potato starch [6]. PEF has been shown to be effective in the extraction of bioactive compounds (mainly betalains) from beetroot, increasing the extraction yield [7]. The greatest increase in the content of betalain compounds in the red beet extract was noted when an electric field at 4.38 kV/cm was applied [7]. The increase in the extraction rate of polyphenols from olive leaves was also observed by using high-voltage electrical discharges (HVED) as a green technology [8]. HVED parameters included different green solvents (water, ethanol), treatment times (3 and 9 min), gases (nitrogen, argon), and voltages (15, 20, 25 kV). The highest yield of phenolic compounds was obtained for the sample treated with argon/9 min/20 kV/50% (3.2 times higher as compared to conventional extraction (CE)). In general, HVED presents an excellent potential for phenolic compound extraction for further application in functional food manufacturing [8]. 

Valorisation of waste and by-products is the topic of the paper by Løvdal et al. [9]. It provides an overview of tomato production in Europe and the strategies employed for processing and valorisation of tomato side streams and waste fractions. Special emphasis was put on the four tomato-producing countries Norway, Belgium, Poland, and Turkey. These countries are very different with regard to their climatic preconditions for tomato production and volumes produced and represent the extremes among European tomato producing countries. 

Osmotic dehydration and drying of berries were the objective of papers by Nowacka et al. [10] and Stamenković et al. [11]. In the paper by Nowacka et al. [10], osmotic dehydration of cranberries was combined with blanching, ultrasound, and vacuum application. Unconventional pretreatment of cranberries caused a significant increase of osmotic dehydration effectiveness. Cranberries subjected to combined treatment, in particular to ultrasounds, had comparable or higher polyphenolic, anthocyanin, and flavonoid content than a blanched tissue subjected to osmotic dehydration alone. Taking into account the evaluated physical and chemical properties of dehydrated cranberries and the osmotic dehydration process, it has been concluded that the best combined pretreatment method was a 20 min sonication followed by a 10 min lowered pressure treatment. In the paper by Stamenković et al. [11], the effectiveness of convective drying of Polana raspberries was compared to freeze-drying, which allows producers to obtain products of high quality but also with high cost. The authors concluded that convective drying of Polana raspberry with air temperature of 60 °C and air velocity of 1.5 m·s^−1^, may be considered as a sufficient alternative to freeze-drying [11]. 

Another emerging nonthermal technology studied on fruits and vegetables is high-pressure processing, with the aim of better preserving nutritional and organoleptic properties. In fact, the results presented in the paper by Paciulli et al. [12] revealed the mild impact of high-pressure treatments on the organoleptic properties of blueberries along with better texture and colour maintenance. The effects of ultra-high pressure (UHP) and thermo-sonication (TS) were also tested on quality of mango juice [13]. Both treatments had minimal effects on the total soluble solids, pH, and titratable acidity of mango juice. The residual activities of three enzymes (polyphenol oxidase, peroxidase, and pectin methylesterase), antioxidant compounds (vitamin C, total phenolics, mangiferin derivatives, gallotannins, and quercetin derivatives) and antioxidant activity sharply decreased with the increase in the temperature of the TS treatment. Nevertheless, the UHP treatment retained antioxidants and antioxidant activity at a high level. The UHP process is apparently superior to TS in bioactive compound and antioxidant activity preservation. Therefore, the mango juice products obtained by ultra-high-pressure processing might be more beneficial to health [13]. 

In the paper by Wiktor et al. [14], instead, the effect of pulsed light treatment with different fluence was studied on a gallic acid aqueous solution—as a model system of phenolic abundant liquid food matrices. It was demonstrated that pulsed light can modify the optical properties of gallic acid and cause reactions and degradation of gallic acid. However, application of pulsed light did not significantly alter the overall quality of the model gallic acid solution at low fluence levels. Cluster analysis revealed that below 3.82 J/cm^2^, changes in gallic acid were minimal, and this fluence level could be used as the critical level for food process design aiming to minimize nutrient loss. 

Finally, Tomas-Egea et al. [15] studied the importance of process control in the industry, which requires fast, safe, and easily applicable methods. In this sense, the use of dielectric spectroscopy in the microwave range can be a great opportunity to monitor processes in which the mobility and quantity of water is the main property to produce a high-quality and safe product, such as candying of fruits. They demonstrated that the use of dielectric properties in γ-dispersion at relaxation frequency allowed us not only to monitor the osmotic drying and hot-air-drying processes of the apple candying, but also to predict the supersaturation state of the liquid phase until vitrification.
